# The Abscisic Acid Receptor Gene *StPYL8-like* from *Solanum tuberosum* Confers Tolerance to Drought Stress in Transgenic Plants

**DOI:** 10.3390/antiox13091088

**Published:** 2024-09-05

**Authors:** Panfeng Yao, Chunli Zhang, Chao Sun, Yuhui Liu, Zhen Liu, Jia Wei, Xinglong Su, Jiangping Bai, Junmei Cui, Zhenzhen Bi

**Affiliations:** 1 State Key Laboratory of Aridland Crop Science, Gansu Agricultural University, Lanzhou 730070, China; yaopf@gsau.edu.cn (P.Y.); zhangchunl@st.gsau.edu.cn (C.Z.); sunc@gsau.edu.cn (C.S.); lyhui@gsau.edu.cn (Y.L.); liuzhen@gsau.edu.cn (Z.L.); wej@st.gsau.edu.cn (J.W.); suxl@st.gsau.edu.cn (X.S.); baijp@gsau.edu.cn (J.B.); cuijm@gsau.edu.cn (J.C.); 2 College of Agronomy, Gansu Agricultural University, Lanzhou 730070, China

**Keywords:** potato, drought tolerance, *StPYL8-like*, expression analysis

## Abstract

Pyrabactin resistance 1-like (PYL) proteins are abscisic acid (ABA) receptors that play a crucial role in the plant’s response to adverse environmental conditions. However, as of yet, there is limited research on the role of PYL proteins in potato. In this study, a potato *PYL* gene, *StPYL8-like*, was identified through transcriptome analysis under drought stress. Molecular characterization revealed that the StPYL8-like protein possesses a highly conserved PYL family domain. Evolutionary analysis demonstrated that StPYL8-like protein clusters with various PYL proteins are involved in stress responses across different species. Functional assays showed that *StPYL8-like* robustly responds to different abiotic stresses, including drought and ABA treatment. Furthermore, the transient and stable expressions of *StPYL8-like* in tobacco enhanced their drought resistance, leading to increased plant height, leaf number, and fresh weight, as well as an improved root system. Transgenic tobacco carrying the *StPYL8-like* gene exhibited lower malondialdehyde (MDA) levels and higher proline accumulation and antioxidant enzyme activity compared to wild-type plants under drought conditions. Moreover, *StPYL8-like* upregulated the expression of stress-responsive genes (*NtRD29A*, *NtLEA5*, *NtP5CS*, *NtPOD*, *NtSOD*, and *NtCAT*) in transgenic plants subjected to drought stress. Collectively, these findings highlight the positive regulatory role of the *StPYL8-like* gene in enhancing potato plants’ response to drought stress.

## 1. Introduction

The growth and development of plants are frequently impacted by a variety of abiotic stresses, including drought, low temperature, high salinity and so on. Abiotic stress poses a significant latent threat to agricultural production, often resulting in pervasive reductions in crop yield. In response to abiotic stress, plants must orchestrate a range of physiological and biochemical responses, in addition to gene regulation, to effectively acclimate to adverse environmental conditions [1,2]. Gene regulation is intricately linked to stress-induced hormonal signaling pathways. Studies have demonstrated the critical involvement of ABA as a stress hormone in mediating plant responses to abiotic stressors [3,4]. It is crucial to conduct in-depth research on the ABA signaling pathway to improve plant adaptability to stressful environments.

Previous studies have identified the three fundamental components of the ABA signaling pathway: the ABA receptor PYL protein, the negative regulatory factor 2C protein phosphatase (PP2C), and the positive regulatory factor SNF1-related protein kinase 2 (SnRK2). These components collectively establish a dual-negative regulatory system, known as PYL-PP2C-SnRK2, which governs ABA signaling and subsequent responses [5,6]. Under normal circumstances, plants maintain low ABA levels, leading to the dimeric form of PYL that is unable to interact with PP2C. Consequently, PP2C exhibits high phosphatase activity, binding to SnRK2 to suppress its kinase function, thereby impeding downstream transcription-factor phosphorylation and gene expression within the ABA signaling pathway. However, in response to stressors like drought, ABA levels surge, facilitating the binding of ABA to and inducing a conformational change in PYL from its dimeric to its monomeric form. The monomeric PYL-ABA complex then associates with PP2C, forming a ternary complex that obstructs the active site of PP2C’s phosphatase activity. This interaction releases SnRK2 activity, enabling the phosphorylation of downstream targets and the subsequent regulation of stomatal closure and resistance mechanisms [7,8]. In essence, the ABA receptor protein PYL functions upstream of the PYL-PP2C-SnRK2 regulatory system within the ABA signaling pathway, crucially involved in ABA signal recognition, PP2C inhibition, and the initiation of ABA signaling transduction.

As a stress hormone, ABA content in plants rapidly elevates under abiotic-stress conditions, particularly drought and salt stress. In recent years, the role of ABA receptors in plant responses to abiotic stress has been gradually elucidated. To date, researchers have conducted functional analyses on 35 PYL proteins across 13 species, revealing the pivotal role of PYL in plant responses to abiotic stress [9], root development [10], fruit ripening [11], and various physiological processes. Primarily, PYL’s function lies in mediating signal transduction in plant responses to abiotic stress, with divergent mechanisms of action observed. For instance, Arabidopsis *PYL5* bolsters drought resistance by directly inhibiting type A PP2Cs [12]; wheat *PYL4* enhances water-use efficiency and drought tolerance by modulating stomatal conductance and elevating photosynthetic activity in transgenic plants [13]; rice *PYL3/10* heightens seed sensitivity to ABA during germination and early seedling stages, thereby enhancing adaptability to stress factors [14]; and maize *PYL8/9/12* enhances drought tolerance in transgenic plants through increased proline accumulation [15]. Furthermore, investigations on *PYL* gene function in various plant species such as grapes [16], tomatoes [17], rapeseed [18], strawberries [19], and apples [20] have demonstrated that modulating the expression of this gene family can enhance drought resistance in plants. In essence, the *PYL* gene significantly contributes to plant responses to drought stress, with its regulatory potential offering a means to enhance plant resilience, albeit through diverse mechanisms of action.

Potato (*Solanum tuberosum* L.) plays a critical role in ensuring global food security as the fourth-largest staple crop globally. It is primarily grown in northwestern China, where water scarcity frequently induces drought stress in potatoes, consequently exerting a significant influence on both local and national commercial potato production [21]. Consequently, there is a growing emphasis on exploring and characterizing drought-resistant genes in potato to enhance stress tolerance. Some progress has also been made in the functional study of key genes in the ABA signaling pathway for potato drought tolerance. For instance, Bai et al. [22] identified the *SnRK* family genes in potatoes, and Yao et al. [23] further validated the drought-resistance function of this gene family. Wang et al. [24] conducted a comprehensive screening of the potato *PP2C* gene family at the genome level and observed the response of each gene to abiotic stress. Garia et al. [25] discovered that *StPP2Ac2b* plays a positive regulatory role in tuber formation in creeping stems. Moreover, Gui et al. [26] recently identified the *PYL* family genes in potatoes and assessed the response of each gene to various abiotic stresses. In the initial phase of this study, comprehensive whole-genome profiling of the potato *PYL* gene family was carried out, alongside the assessment of the expression profiles of individual *PYL* genes under drought conditions. Subsequently, *StPYL8-like*, exhibiting a pronounced response to drought stress, was chosen as the target for further exploration, and its role in drought resistance mechanisms was investigated. Then, the drought-resistance function of *StPYL8-like* was verified through transient and stable transformation methods. The outcomes of this investigation advance and diversify research on the ABA signaling pathway in potatoes, offering insights for exploring the molecular regulatory network of potato drought tolerance and enhancing the molecular breeding of drought-resistant potato cultivars.

## 2. Materials and Methods

### 2.1. Plant Material and Growth Conditions

The potato cultivars “Qingshu 9” (QS9) and the tobacco variety NT12 were conserved at the State Key Laboratory of Aridland Crop Science of Gansu Agricultural University. Potato plants were cultivated in an artificial growth chamber under a light regimen of 16 h of illumination and 8 h of darkness, maintained at a temperature of 22 ± 2 °C, with a relative humidity of 60%. Tobacco seedlings were cultured in a greenhouse with a temperature of 25 ± 2 °C, a light cycle of 12 h of light and 12 h of darkness, and a relative humidity of 60%. The plant expression vector pCAMBIA1304 and *Agrobacterium* tumefaciens GV3101 were housed within our laboratory.

### 2.2. Isolation and Characterization of StPYL8-like

The potato reference-genome sequence and GFF annotation file were acquired from the potato genome website (http://spuddb.uga.edu/, accessed on 5 March 2022), while all AtPYL protein sequences were sourced from TAIR (https://www.arabidopsis.org/, accessed on 5 March 2022). The StPYL protein sequences were derived through sequence alignment and screening using TBtools. Subsequently, the expression profile of *StPYLs* was analyzed through transcriptome data of potatoes under drought stress. Finally, the *StPYL8-like* gene (Gene ID: PGSC0003DMG400005016) that significantly responds to drought stress was cloned. Following the sequencing outcomes, specific primers were designed for the amplification of the *StPYL8-like* gene (534 bp, GenBank ID: XP_006360709), which was subsequently cloned using potato young-leaf cDNA as a template. The protein sequence encoded by *StPYL8-like* was then subjected to multiple-sequence alignment and phylogenetic analysis using the Cluster X and MEGA software tools (https://app.clusterx.com/, https://www.megasoftware.net/, accessed on 8 April 2022).

### 2.3. Expression Analysis of StPYL8-like

The response of *StPYL8-like* to diverse abiotic stresses was detected using qRT-PCR. Three-week-old potato plants were subjected to stress treatments, including 200 mM mannitol, 100 mM NaCl, 100 µM ABA, 100 µM SA, and 100 µM MeJA. Control conditions consisted of a normal MS medium for drought and salt treatments, while alcohol spraying served as the control for hormone treatments. Samples were collected 0, 1, 3, 6, and 12 h post stress induction, followed by RNA extraction post liquid-nitrogen freezing. Each stress treatment was replicated thrice. The qRT-PCR primers are detailed in Supplementary Table S1.

### 2.4. Promoter cis-Element Analysis

The 2000 bp promoter sequence upstream of the start codon of the *StPYL8-like* gene was extracted using TBtools. Subsequently, the cis-acting elements of the *StPYL8-like* promoter sequence were analyzed utilizing the PlantCARE online tool (http://bioinformatics.psb.ugent.be/webtools/plantcare/html/, accessed on 15 April 2022). Following this, *StPYL8-like* promoter was incorporated into the plant expression vector pBI101-GUS through homologous recombination. The transient transformation of 4-week-old tender tobacco leaves was carried out using *Agrobacterium* tumefaciens GV3101, with samples collected 2 days later for GUS histological staining at 37 °C to assess the activity of the *StPYL8-like* promoter.

### 2.5. Evaluation of Drought Resistance after Transient Transformation of Tobacco

The coding sequence (CDS) of the *StPYL8-like* gene was amplified by PCR using gene-specific primers. The PCR product was subsequently digested with *Bgl* II and *Spe* I restriction enzymes and ligated into the pCAMBIA1304-GUS vector to generate the recombinant plasmid pC1304-StPYL8-like-GUS. The construct was then transformed into *Agrobacterium* tumefaciens GV3101 cells using the heat-shock method. To assess the biological function of the *StPYL8-like* gene, transient transformation was performed in tobacco plants. Tobacco plants grown under normal conditions for approximately 4 weeks were transferred to Hoglund nutrient solution for a 2-day culture period. Following this, GV3101 harboring pC1304-StPYL8-like-GUS was infiltrated into tobacco leaves, with the empty vector pC1304 serving as a negative control. After 2 days of recovery, all the plants were subjected to stress treatment with 20% PEG600 in the Hoglund nutrient solution. GUS staining was carried out after 6 h to confirm successful transformation. Subsequently, various physiological parameters associated with stress responses were measured to evaluate the impact of the *StPYL8-like* gene on drought tolerance in transgenic tobacco plants.

### 2.6. Evaluation of Drought Resistance after Stable Transformation of Tobacco

The recombinant plasmid pC1304-StPYL8-like-GUS was introduced into tobacco T12 through the *Agrobacterium*-mediated leaf-disk transformation method. Subsequently, transgenic plants were cultivated on MS medium and screened for resistant seedlings using 1/2 MS medium supplemented with hygromycin (50 mg L^−1^, *w*/*v*). Positive lines were then confirmed by qRT-PCR and GUS staining. Based on the qRT-PCR results, three transgenic lines exhibiting the highest *StPYL8-like* expression levels were selected. These lines were subsequently subjected to drought stress by cutting them into uniform stem segments and transferring them to MS medium supplemented with 100 mM, 200 mM, and 300 mM mannitol. A control group was maintained under normal MS medium conditions. Following a 30-day drought-stress treatment period, various indicators related to stress response were evaluated.

### 2.7. β-Glucuronidase (GUS) Staining

GUS histological staining was performed as detailed in a previous publication. Transgenic plant leaves were collected and immersed in a reaction solution containing 2 mM 5-bromo-4-chloro-3-indolyl-β-D-glucuronic acid, 50 mM sodium phosphate, 10 mM EDTA, 2 mM ferrocyanide, and 0.1% Triton X-100 at pH 7.0. The samples were then incubated at 37 °C in darkness for 12 h. Subsequently, the stained leaves were subjected to a gradient elution process using 30%, 60%, and 100% ethanol in an 80 °C water bath. Once chlorophyll was completely removed, micrographs were captured using an optical microscope.

### 2.8. Determination of Phenotypic and Physiological Indicators

The proline and malondialdehyde (MDA) contents were quantified following the methodologies outlined in previous studies. Enzyme activities of superoxide dismutase (SOD), peroxidase (POD), and catalase (CAT) were assessed in both transgenic and control plants utilizing commercially available assay kits (BC0170, BC0220, BC0200, and BC0200 from Beijing Solaibao Technology Co., Ltd., Beijing, China). Phenotypic characteristics primarily encompass plant height, stem diameter, and root length. Furthermore, a root scanner was employed for the evaluation of root parameters including total root length, number of root tips, root surface area, and root volume.

### 2.9. Expression Measurement of Stress-Responsive Genes

Total RNA was extracted from tobacco samples subjected to various stress conditions and reverse-transcribed to cDNA. Subsequently, specific primers (Table S1) were employed to quantify the expression levels of stress-responsive genes via qRT-PCR.

### 2.10. Statistical Analysis

The expression analysis and physiological index determination under abiotic stress were both performed for three independent biological replicates with three technical repeats. The mean and standard deviation (SD) were calculated for each treatment based on the collected data from these replicates. A one-way analysis of variance (ANOVA) was conducted using SPSS v19.0 (SPSS Inc., Chicago, IL, USA) to assess the variability among the three overexpression lines (OE) and control plants, with statistical significance defined as *p* < 0.05 or *p* < 0.01.

## 3. Results

### 3.1. Molecular Characterization Analysis of StPYL8-like

The whole-genome identification results revealed the presence of 20 *PYL* genes in potatoes, among which three genes exhibited two different transcripts (Table S2). All PYL proteins contain the distinctive conserved domains characteristic of this protein family (Figure S1). Subsequently, the response of all *StPYL* genes to drought stress was evaluated through the transcriptome analysis of ‘QS9’ potato under drought conditions, revealing distinct expression patterns among *PYL* family members under such stress. Notably, *StPYL16*, displaying a significant response to drought stress, was singled out for further investigation (Figure S2). Nevertheless, the sequence was submitted to the NCBI database a few years ago and named *StPYL8-like* (GenBank ID: XP_006360709). To ensure coherence, we ultimately labeled the *PYL* sequence under investigation in this study *StPYL8-like*.

**Figure 1:**
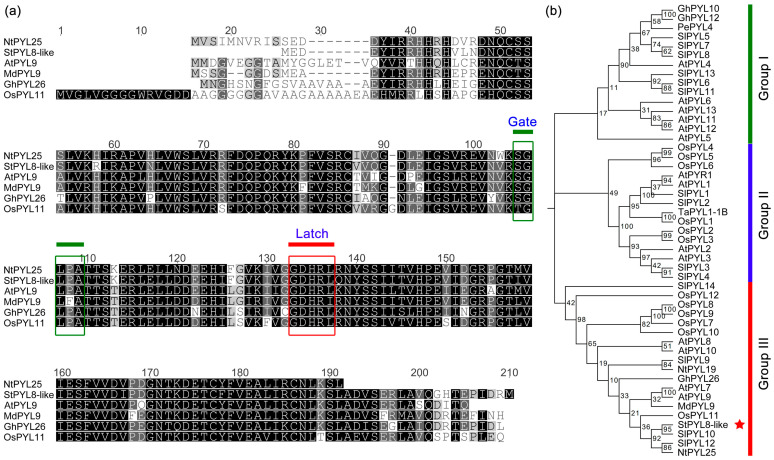
Analysis of the structure of *StPYL8-like* protein. (**a**) Sequence alignment of *StPYL8-like* with other PYL proteins. Gate and Latch conserved domains are indicated; (**b**) Phylogenetic relationship of *StPYL8-like* with other PYL proteins. The red asterisk represents potato *StPYL8-like* protein.

The *StPYL8-like* gene comprises a 534 bp open reading frame that encodes a protein consisting of 177 amino acids. Blastx analysis revealed that StPYL8-like shares sequence identities with AtPYL9 and AtPYL8 of 78% and 77% in Arabidopsis, respectively. Subsequent sequence alignment demonstrated a high similarity between the StPYL8-like protein sequence and the conserved domains of PYL proteins found in Arabidopsis, apple, cotton, and rice. Moreover, typical Gate and Latch motifs, which are key features of the PYL family proteins, were identified (Figure 1a). Additionally, phylogenetic analysis placed StPYL8-like in subgroup III, where it clustered with most PYL proteins involved in abiotic stress regulation, such as MdPYL9, GhPYL26, and OsPYL7/10/11 (Figure 1b). This suggested that StPYL8-like may possess similar biological functions to those proteins.

### 3.2. StPYL8-like Is Strongly Induced by Drought Treatment

**Figure 2:**
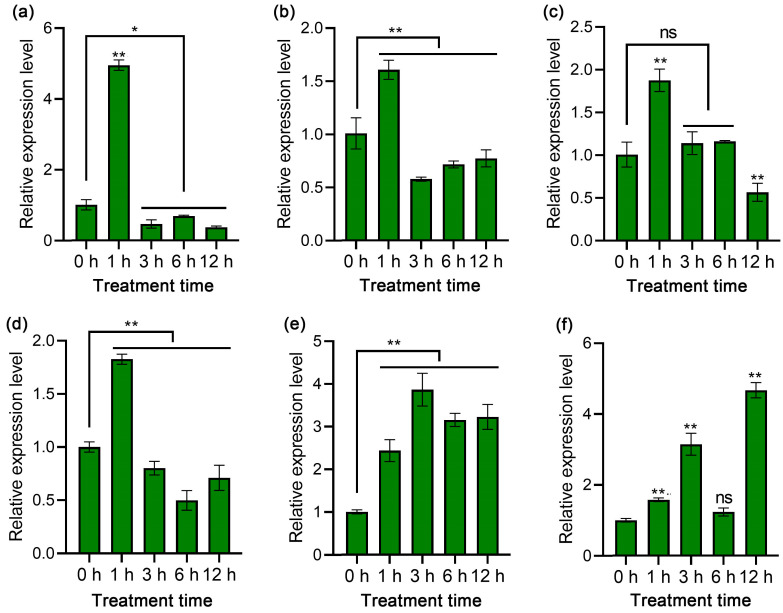
The relative expression level of the *StPYL8-like* gene under abiotic-stress conditions. (**a**–**f**) represent 200 mM mannitol, 100 mM NaCl, 100 µM ABA, 100 µM SA, and 100 µM MeJA stress, respectively. Data represent the means ± SD of three replicates. * and ** indicate significant difference at *p* < 0.05 and *p* < 0.01 levels, respectively. ns indicates that the difference is not significant. The horizontal line above the column indicates that the data below the line have the same level of significance difference compared to the data at 0 h.

To explore the reaction of *StPYL8-like* to a range of abiotic stresses, such as drought, salt, 4 °C, and ABA and SA treatments, experiments were conducted (Figure 2). In general, the *StPYL8-like* gene displays diverse expression patterns in response to the different abiotic-stress treatments. The findings revealed that in QS9, *StPYL8-like* demonstrated a pattern of initial increase followed by decrease across multiple stressors. Specifically, under drought, salt, 4 °C, and ABA stimuli, *StPYL8-like* exhibited a notable rise to its peak value after 1 h of stress, followed by a gradual decline after 3 h. Conversely, under SA and MeJA stimuli, a continuous upward trajectory was observed.

### 3.3. Transient Transformation of StPYL8-like Improves Drought Tolerance of Transgenic Tobacco

**Figure 3:**
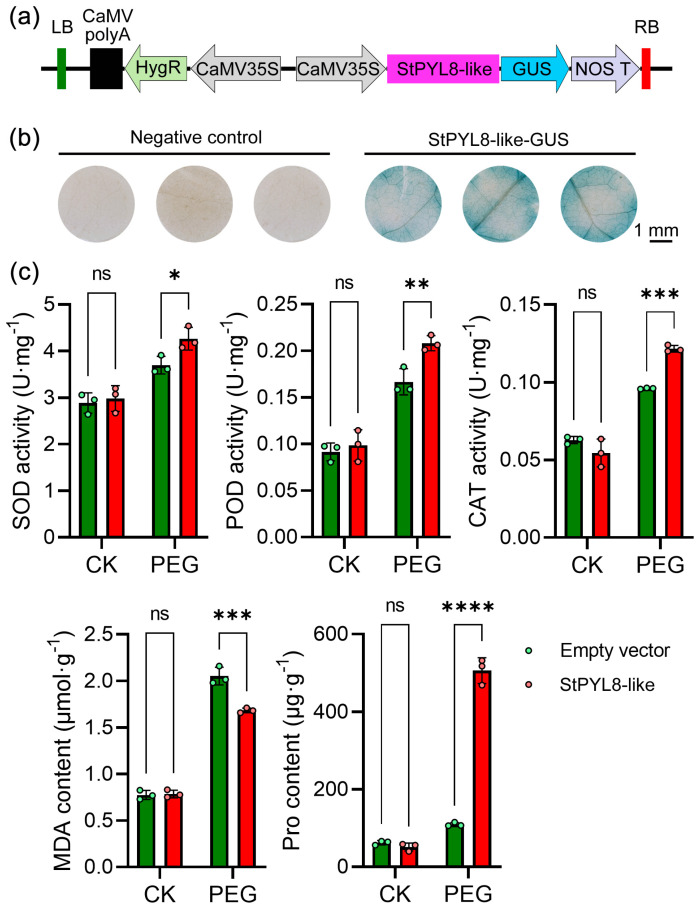
Identification of drought resistance in tobacco after transient transformation of the *StPYL8-like* gene. (**a**) Schematic diagram of the *StPYL8-like* gene plant-overexpression vector; (**b**) GUS histochemical staining of *StPYL8-like* transgenic plants; (**c**) Determination of physiological indexes related to stress. CK and PEG represent normal Hogland nutrient solution and Hogland nutrient solution containing 20% PEG6000, respectively. *, **, ***, and **** indicate significant difference at *p* < 0.05, *p* < 0.01, *p* < 0.001, and *p* < 0.0001 levels, respectively. ns indicates that the difference is not significant.

To preliminarily investigate the function of *StPYL8-like*, we transiently expressed it in tobacco and exposed the plants to drought stress (Figure 3a). Initially, GUS staining was conducted on the transgenic plants, revealing a distinct blue color in *StPYL8-like*-transformed leaves as opposed to slightly yellow control leaves, indicating the successful integration of the target gene (Figure 3b). Furthermore, to elucidate the effects of *StPYL8-like* on transgenic plants, we evaluated the activities of antioxidant enzymes (SOD, POD, and CAT) closely associated with plant stress responses, along with the accumulation levels of MDA and Pro (Figure 3c). Our findings demonstrated that the overexpression of *StPYL8-like* resulted in a substantial increase in Pro accumulation in transgenic plants under drought-stress conditions while decreasing MDA levels. Conversely, no significant differences were observed between the two indicators under normal conditions. The activity of antioxidant enzymes followed a similar trend. Under normal circumstances, there was a certain variance in antioxidant enzyme activity between control and transgenic plants, albeit not statistically significantly; however, under drought-stress conditions, the activity of all three antioxidant enzymes in transgenic plants exhibited a noteworthy increase compared to the control.

### 3.4. Generation of Stable Transgenic Tobacco

**Figure 4:**
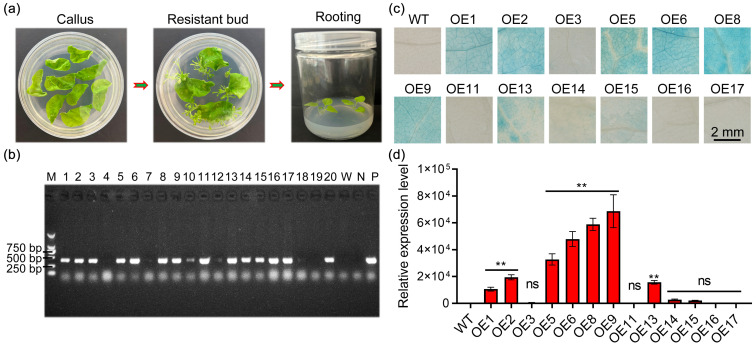
Positive identification of *StPYL8-like* transgenic tobacco. (**a**) The process of transforming *StPYL8-like* into tobacco; (**b**) PCR molecular identification of positive *StPYL8-like* transgenic lines; (**c**) GUS histochemical staining of *StPYL8-like* transgenic lines and control plants; (**d**) Analysis of expression level of *StPYL8-like* in transgenic lines. ** represents significant differences between transgenic lines and WT at *p* < 0.01. ns indicates that the difference is not significant. The horizontal line above the column indicates that the data below the line have the same level of significance difference compared to the data at WT.

To further explore the role of *StPYL8-like* in response to drought stress, we established an overexpression of *StPYL8-like* in tobacco via an *Agrobacterium*-mediated leaf-disc transformation technique (Figure 4a). Subsequent hygromycin selection yielded 20 transgenic plants exhibiting resistance. Following RT-PCR analysis, the presence of the vector tag gene was confirmed in 13 out of the 20 resistant plants (Figure 4b). This finding was consistent with positive GUS histological staining in seedlings (Figure 4c). Subsequently, the quantitative assessment of *StPYL8-like* expression levels in each positive plant was performed using qRT-PCR, leading to the selection of three transgenic lines (OE-6, OE-8, and OE-9), characterized by high *StPYL8-like* expression levels, for further investigation (Figure 4d).

### 3.5. Overexpression of StPYL8-like Increases Tobacco Drought Tolerance

**Figure 5:**
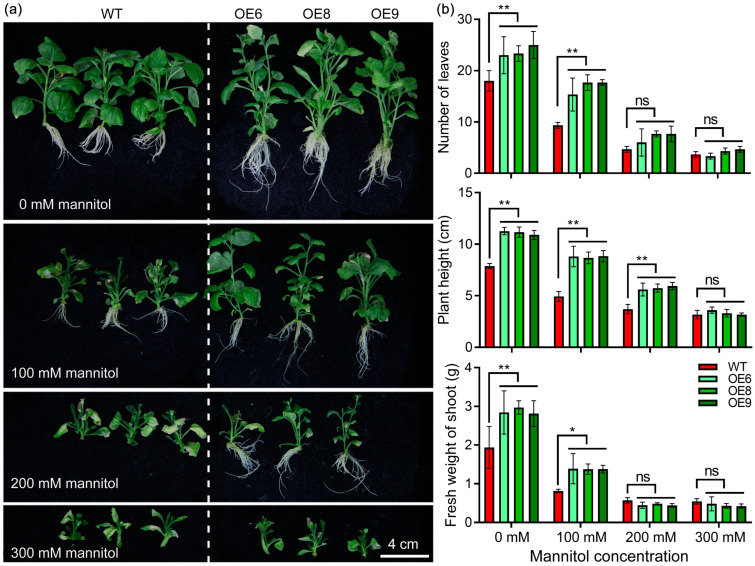
Drought-resistance function of *StPYL8-like* after stable transformation of tobacco. Transgenic tobacco-stem segments with consistent growth were transferred to MS medium as well as MS medium supplemented with 100 mM, 200 mM, and 300 mM mannitol for 30 d to stress, followed by the measurement of various phenotypic traits. (**a**) Phenotypic identification of plants under drought stress; (**b**) Statistics of leaf number, plant height and fresh weight of plants under normal and drought stress. * and ** indicate significant difference at *p* < 0.05 and *p* < 0.01 levels, respectively. ns indicates that the difference is not significant. The horizontal line above the column indicates that the data below the line have the same level of significance difference compared to the data at WT.

To determine the drought-resistance function of *StPYL8-like*, transgenic tobacco and control plants were subjected to drought-stress treatments. Initially, images of each plant genotype were captured under stress conditions for visual assessment. The results indicated discernible disparities in the growth patterns between transgenic and control strains under normal conditions, with transgenic plants displaying superior growth performance. Upon exposure to stress, the growth of all plants was impeded, with control plants exhibiting a more pronounced inhibition compared to transgenic plants. Furthermore, this discrepancy magnified with escalating stress severity. A notable observation was that transgenic plants maintained normal root growth at a concentration of 200 mM mannitol, whereas the root growth of control plants was completely suppressed (Figure 5a).

To more specifically illustrate the impact of *StPYL8-like* overexpression on the growth of transgenic plants, detailed measurements were conducted on parameters including leaf number, plant height and fresh weight (Figure 5b). The results indicated that under normal conditions, the *StPYL8-like* transgenic plants exhibited significantly higher leaf numbers, plant heights, and fresh weights compared to control plants, suggesting that the overexpression of *StPYL8-like* may contribute to both plant development and enhanced drought resistance in transgenic plants. This difference was even more pronounced under 100 mM mannitol stress conditions. When the root growth of wild-type plants was inhibited (200 mM mannitol), no significant differences were observed in leaf number and fresh weight compared to transgenic plants; however, the plant height remained notably lower in wild-type plants. Under 300 mM mannitol conditions, no significant variations were detected in the aforementioned parameters.

**Figure 6:**
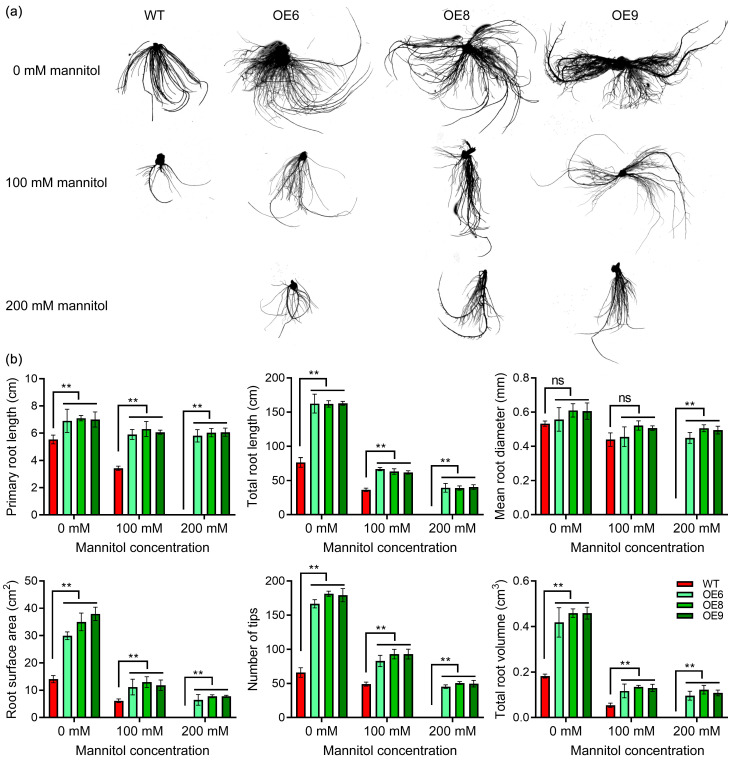
Identification of root phenotype of *StPYL8-like* transgenic plants under stress conditions (**a**) Root-scanning diagram of each plant under different treatment conditions; (**b**) Determination of root system-related indexes of various plants under different treatment conditions. ** represents significant differences between transgenic lines and WT at *p* < 0.01. ns indicates that the difference is not significant. The horizontal line above the column indicates that the data below the line have the same level of significance difference compared to the data at WT.

The aforementioned findings demonstrated that the overexpression of *StPYL8-like* has a substantial impact on the growth of transgenic plant roots. Subsequently, we conducted a comprehensive assessment of root phenotypes and associated metrics across different plant strains subjected to varying stress conditions. Morphologically, the root system of *StPYL8-like* transgenic plants exhibited superior development compared to control plants under both normal and stress conditions (Figure 6a). These observations were substantiated by the evaluation of pertinent metrics. Specifically, parameters such as primary root length, total root length, root area, number of root tips, and total root volume displayed a consistent pattern. Under normal circumstances, the metrics for transgenic plants were markedly higher than those for control plants, whereas under stress conditions, all line indicators experienced inhibition; however, the discrepancy persisted, peaking at 200 mM mannitol (Figure 6b). Notably, there was no significant alteration in root diameter pre- and post-stress during standard root growth.

**Figure 7:**
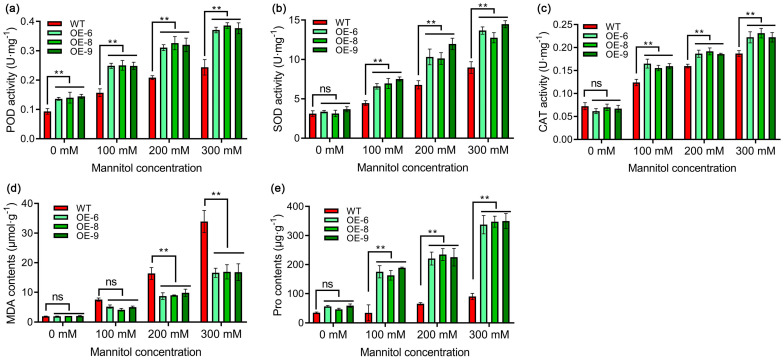
Determination of stress-related physiological index contents in different plants under different treatment conditions. (**a**) POD activity; (**b**) SOD activity; (**c**) CAT activity; (**d**) MDA content; (**e**) Pro content. WT represents wild tobacco. OE-6, OE-8 and OE-9 represent three transgenic lines. ** indicates significant difference at *p* < 0.01 level. ns indicates that the difference is not significant. The horizontal line above the column indicates that the data below the line have the same level of significance difference compared to the data at WT.

Finally, the activity of three antioxidant enzymes associated with anti-stress was further measured. As showed in the figure, under normal conditions, the POD activity of transgenic plants was significantly higher than that of the control, whereas the SOD and CAT activities did not display significant deviations from the control. Conversely, when subjected to stress conditions, the activities of all three enzymes followed a similar trend of alteration, with enzyme levels in transgenic plants markedly surpassing those in control plants. Notably, this disparity widened concomitantly with escalating stress intensity. Moreover, two key physiological indicators closely associated with drought stress, MDA and Pro, were assessed (Figure 7). While the overexpression of *StPYL8-like* led to alterations in the growth phenotype of transgenic plants under normal conditions, there was no discernible impact on the accumulation of MDA and Pro compared to the control group. Specifically, no significant differences were observed between the transgenic plants and the control group under normal conditions. However, when exposed to stress conditions of 200 mM and 300 mM mannitol, the MDA levels in each transgenic strain were markedly lower than those in the control plants, with a noticeable concentration-dependent effect. Similarly, under stress conditions, the Pro levels in transgenic plants were considerably higher than those in the control plants, and this disparity intensified with the severity of the stress.

### 3.6. Stress-Related Gene Expression in Transgenic Tobacco Plants under Drought Stress Mediated by StPYL8-like

**Figure 8:**
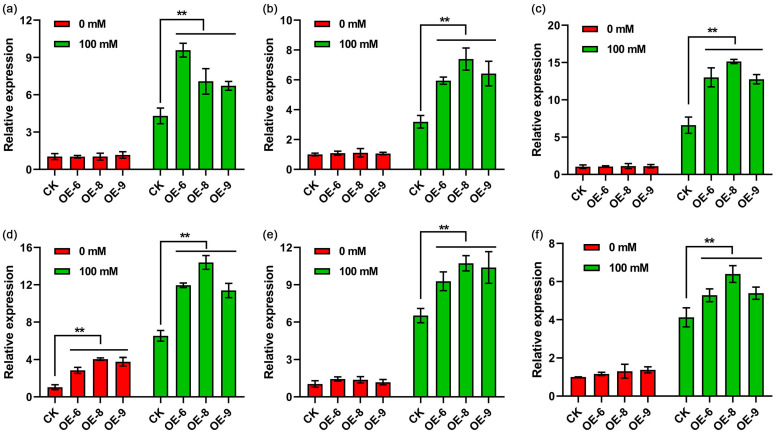
Expression analysis of stress-related genes in transgenic plants and wild-type plants. (**a**–**f**) represents *NtRD29A*, *NtLEA5*, and *NtP5CS*, *NtPOD*, *NtSOD*, *NtCAT* genes, respectively. ** represents significant differences between transgenic lines and WT at *p* < 0.01. CK represents wild tobacco. OE-6, OE-8 and OE-9 represent three transgenic lines. 0 mM and 100 mM represent normal MS medium and MS medium containing 100 mM mannitol, respectively. The horizontal line above the column indicates that the data below the line have the same level of significance difference compared to the data at CK.

To investigate the potential mechanism underlying the enhancement of drought tolerance in transgenic plants expressing *StPYL8-like*, we analyzed the expression patterns of stress-related genes in both *StPYL8-like* transgenic lines and control plants under normal and drought conditions. Our findings revealed that upon exposure to drought stress, the transcript levels of various stress-related genes, such as *NtRD29A*, *NtLEA5*, *NtP5CS*, *NtPOD*, *NtSOD*, and *NtCAT*, were markedly elevated in *StPYL8-like* transgenic plants compared to the control plants (Figure 7). Interestingly, no significant differences were observed in gene expression levels between *StPYL8-like* transgenic plants and control plants under normal growth conditions, except for the *NtPOD* gene. Importantly, under drought-stress conditions, the expression levels of all identified genes in both wild-type and transgenic plants exhibited a substantial increase relative to normal conditions (Figure 8). These results suggest that the overexpression of *StPYL8-like* could potentially modulate the expression of these stress-related genes either directly or indirectly, thereby bolstering the drought resistance of transgenic plants.

## 4. Discussion

The plant hormone ABA plays a pivotal role in regulating plant growth, development, and responses to stress [27]. Drought stress typically results in heightened intracellular ABA synthesis, with the ABA receptor protein PYL playing a central role in the ABA-mediated signaling pathway that governs plant drought-resistance mechanisms [28]. This current study successfully cloned the *StPYL8-like*, a member of the *PYL* gene family, from potato. Subsequently, following a comprehensive molecular characterization, we confirmed its regulatory impact on drought resistance by assessing transient and stable expression in transgenic tobacco plants.

While ABA plays a pivotal role in this process, it was not until 2009 that researchers unearthed PYL in an investigation on a synthetic ABA agonist known as pyranose, designating PYL as an ABA receptor [29]. Afterwards, extensive research endeavors have been directed towards the screening and identification of PYL family proteins across diverse plant species. In Arabidopsis, 14 PYL proteins have been categorized into three subfamilies based on phylogenetic analysis and structural and functional characterization [30]. Discrepancies in the number of *PYL* family genes have been unveiled among different plant species through whole-genome identification. For instance, there are 15 in tomato [31], 40 in cotton [32], 14 in poplar [33], 8 in grapes [16], 38 in wheat [34], and 13 in rice [35]. Despite variations in the quantity of *PLY* family genes across species, the clustering patterns of these genes exhibit substantial consistency among plants. The classification of PYL family members into three subfamilies in various species indicates a relatively conserved function of PYL proteins throughout the evolutionary process. Recent investigations, utilizing transcriptome data from drought-stressed potato, have pinpointed the *StPYL8-like* gene as significantly responsive to drought stress. Further phylogenetic analysis has revealed that StPYL8-like protein clusters with PYLs are implicated in the abiotic stress response in organisms such as Arabidopsis, rice, and cotton (Figure 1). This finding underscores the potential significance of *StPYL8-like* in mediating the potato’s response to drought stress.

Numerous studies have validated its function in enhancing plant drought tolerance. For instance, the overexpression of *AtPYR1* and *AtPYL1*/2/3/8/9 notably boosted the drought tolerance of transgenic Arabidopsis [36]. Comparable outcomes have likewise been observed in other plant species like maize [15], rice [14], poplar [37], wheat [13], and cotton [38]. Wheat *PYL4* demonstrates the ability to enhance water-use efficiency and drought resistance by reducing stomatal aperture and increasing photosynthetic activity in transgenic plants [13]. The overexpression of rice *PYL3/10* [14,39] and cotton *PYL10/12/26* [38] heightens seed sensitivity to ABA during the germination and seedling stages, thereby enhancing plant adaptability to abiotic stress. Maize *PYL3/9/10/13* enhances the drought-stress tolerance of transgenic plants by elevating proline accumulation [15]. Research on *PYL* in crops such as grapes [16], tomato [17], rapeseed [18], strawberries [19], and apple [20] has also exhibited that upregulating this gene family can enhance plant resistance to abiotic stress. This investigation identifies *StPYL8-like*, a member of the *PYL* gene family, through transcriptome analysis under early drought stress, potentially participating in the potato response to drought stress. Similar to previously studied *PYL* genes, the overexpression of *StPYL8-like* mitigated damage to drought-stressed transgenic plants and improved their growth status under stress conditions. Moreover, the response to ABA results showed that the expression level of the *StPYL8-like* gene was significantly affected after 1 h of stress, and the expression trend was almost identical to that under drought, salt, and low-temperature stress. These findings suggested that the *StPYL8-like* gene may be involved in modulating abiotic stress responses through the ABA signaling pathway, not only in drought stress but also potentially in salt and low-temperature stress. There have been similar reports before, such as the *OsPYL10* gene in rice, which simultaneously enhances the survival rate of transgenic plants under drought- and low-temperature stress conditions [39]; the grape *VaPYL4* gene simultaneously enhances the tolerance of transgenic plants to three abiotic stresses: drought, salt, and low temperature [40]; and the *TaPYL1-1B* gene in wheat not only enhances the drought resistance of transgenic plants but also increases field yield [41]. These collective results highlight the multi-faceted role of ABA receptor PYL in plants, and whether the *StPYL8-like* gene also has the function of regulating multiple signal pathways needs further study and confirmation.

When plants are exposed to drought stress, the equilibrium between reactive-oxygen-species production and clearance in cells is disrupted, leading to an accumulation of reactive oxygen species, resulting in oxidative damage and eventual cell death [42]. This phenomenon has been extensively researched and confirmed in various plant species. In response to drought stress, plants typically upregulate the transcription levels of antioxidant enzyme genes such as *SOD*, *POD*, and *CAT*. Consequently, the activity of SOD/CAT/POD enzymes increases, ultimately facilitating the elimination of reactive oxygen species [43,44]. These response mechanisms are pivotal in enhancing plant resilience to drought stress. This study found that under drought-stress conditions, the expression levels of *SOD/CAT/POD* genes and the activities of SOD/CAT/POD enzymes were significantly elevated in *StPYL8-like* transgenic plants as compared to the control plants (Figure 3 and Figure 8). Notably, under normal growth conditions, the expression of the *NtPOD* gene in *StPYL8-like*-overexpressing plants exceeded that in control plants. The reduced MDA content in transgenic plants lends further support to this observation (Figure 7), indicating a decrease in oxidative damage. The scavenging system for reactive oxygen species is crucial for plants to combat drought stress, involving a variety of enzymatic and non-enzymatic scavengers. These enzymes work collectively to mitigate the deleterious effects of reactive oxygen species [45,46]. MDA, a product of lipid oxidation, serves as a marker of oxidative damage in plants [47]. Proline, an osmolyte present in plant cytoplasm, plays a critical role in maintaining membrane and protein structure, eliminating reactive oxygen species, and minimizing photodamage to chloroplast thylakoid membranes [48]. In this study, the overexpression of *StPYL8-like* led to an elevation in free proline content in transgenic plants under drought stress, accompanied by the upregulation of the key gene *NtP5CS* involved in proline synthesis (Figure 8). Plants possess two pathways for proline synthesis: the glutamate pathway and the ornithine pathway, with the former predominantly active under osmotic stress. In the glutamate pathway, glutamate (Glu) is converted by pyrroline-5-carboxylic acid synthase (P5CS) into glutamyl semialdehyde (GSA), which cyclizes spontaneously to form pyrroline-5-carboxylic acid (P5C). P5C is then converted to proline through the action of pyrroline-5-carboxylic acid reductase (P5CR) [49,50]. The *P5CS* gene, serving as the primary rate-limiting enzyme in this synthesis pathway, is crucial for proline biosynthesis [51,52]. Therefore, the findings of this study suggest that *StPYL8-like* may enhance the drought tolerance of transgenic plants by influencing proline synthesis, necessitating further experimental investigations to elucidate its molecular mechanisms. Furthermore, prior investigations have indicated that *PYL* genes may be involved in plant growth and development or contribute to plant drought resistance through the modulation of growth and development processes. For instance, the overexpression of *GhPYL10/12/26* genes in cotton has been shown to improve drought resistance in transgenic plants, as well as promote enhanced root-system development under normal growth conditions [38]. Arabidopsis *PYL9* not only enhances drought resistance by reducing water evaporation but also by promoting leaf senescence in old leaves and suppressing growth in young tissues under severe drought stress [10]. Intriguingly, parallel findings were observed in our study. Specifically, under standard growth conditions, transgenic plants with *StPYL8-like* characteristics exhibited superior growth compared to control plants, notably reflected in their more robust root systems. Thus, it is hypothesized that *StPYL8-like* genes could not only boost plant drought resistance through facilitating proline synthesis but could also be involved in modulating plant growth and development. The upregulation of the *StPYL8-like* gene results in transgenic plants with well-developed root systems, enabling them to absorb more water during drought stress, thereby improving drought resistance. Hence, our hypothesis is that the *StPYL8-like* gene could modulate transgenic plant-root development via ABA signaling, concurrently facilitating proline biosynthesis, thereby augmenting the drought-stress tolerance of transgenic plants. In light of this hypothesis, we propose to extend our investigation by screening the interacting proteins of StPYL8-like under drought-stress conditions and evaluating the transcriptional impact of gene overexpression on transgenic plants. Through a holistic approach encompassing physiological, biochemical, transcriptional, and proteomic analyses, we could comprehensively elucidate the molecular mechanisms underlying the involvement of *StPYL8-like* genes in plant drought resistance. Furthermore, our upcoming research endeavors will involve an investigation into the presence of single-nucleotide polymorphisms within the *StPYL8-like* gene across various potato genotypes. Subsequently, we aim to elucidate the potential association between such polymorphisms and drought-resistance levels in different potato genotypes. This comprehensive analysis is poised to enhance our understanding of the biological role of the *StPYL8-like* gene while serving as a valuable resource for the establishment of molecular markers linked to drought resistance in potatoes. Ultimately, these findings are anticipated to significantly advance the molecular breeding efforts targeted at developing drought-tolerant potato cultivars.

## 5. Conclusions

In conclusion, in this research, we successfully cloned a member of the potato *PYL* gene family: *StPYL8-like*. Stress-response assays revealed that *StPYL8-like* exhibited significant responses to a range of stress stimuli, such as drought and ABA. Subsequent functional characterization demonstrated that the overexpression of *StPYL8-like* led to elevated levels of proline accumulation and the enhanced activity of antioxidant enzymes in transgenic plants when exposed to drought stress, ultimately resulting in increased drought-stress tolerance.

## Data Availability

All data are available within the manuscript.

## Supplementary Materials

The following supporting information can be downloaded at: https://www.mdpi.com/article/10.3390/antiox13091088/s1, Figure S1: Analysis of conserved domains of PYL proteins in Arabidopsis (A) and potatoes (B); Figure S2: Expression level analysis of *StPYL* genes under drought stress; Table S1: The sequences of primers were used in the study; Table S2: Molecular characteristics of potato PYL family genes.
