# Supplementary material for: The Abscisic Acid Receptor Gene StPYL8-like from Solanum tuberosum Confers Tolerance to Drought Stress in Transgenic Plants
Source: Antioxidants (Basel). 2024 Sep 5;13(9):1088. doi: 10.3390/antiox13091088 (PMC11428994; doi:10.3390/antiox13091088)
Supplement: Supplementary file 1 [file antioxidants-13-01088-s001.zip › Suplementary figures.pdf]

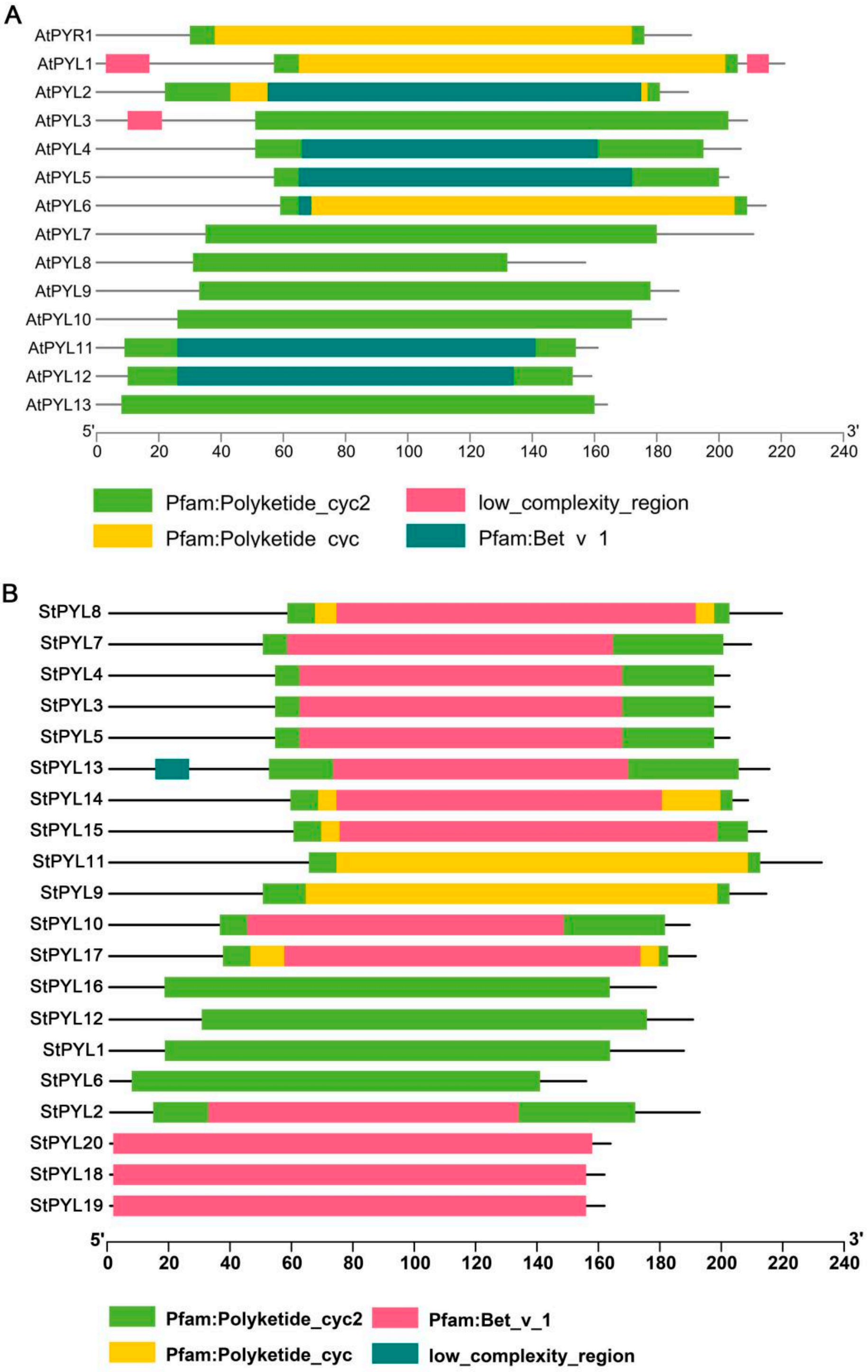

Figure S1 Analysis of conserved domains of PYL proteins in *Arabidopsis* (A) and potatoes (B).

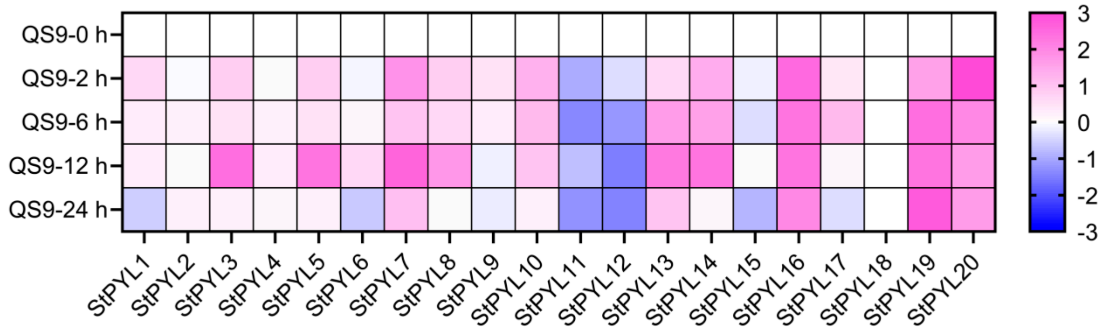

Figure S2 Expression level analysis of *StPYL* genes under drought stress. 3-week-old potato plants were exposed to stress treatment under simulated drought conditions using 200 mM mannitol. Whole plant samples were collected for transcriptome sequencing at 0, 2, 6, 12, and 24 hours post-stress induction. Process the expression level of a gene at 0 hours as "1", and normalize data at other time points relative to the expression level at 0 hours. The intensity of the color indicates the magnitude of the multiplier, with pink colors signifying larger multipliers and blue colors indicating smaller multipliers.
