# Supplementary material for: The Abscisic Acid Receptor Gene StPYL8-like from Solanum tuberosum Confers Tolerance to Drought Stress in Transgenic Plants
Source: Antioxidants (Basel). 2024 Sep 5;13(9):1088. doi: 10.3390/antiox13091088 (PMC11428994; doi:10.3390/antiox13091088)
Supplement: Supplementary file 1 [file antioxidants-13-01088-s001.zip › Suplementary tables.pdf]

Table S1 The sequences of primers were used in the study.

Gene	Sequences	Function
StPYL8-like	F: 5'- ATGGAAGATGAGTACATTAGGAGACAC-3'	Gene clone
	R: 5'- CTACATTCTATCAATAGGTTCGGTATG-3'	
StPYL-like	F: 5'-GGCATGTGCTCAACGACAATCAG-3'	qRT-PCR
	R: 5'-CGCATCTGCTAACGAATGGCTTG-3′	
NtRD29A	F: 5'-TCGGTGTACCAACAGGCATA-3'	
	R: 5'-CCCTTGCTTTGGTGTTGTTT-3'	
NtP5CS	F: 5'-TGGCCCTCCCCGTAATCCAGATTC-3'	
	R: 5'-GATACATTCCCCATGTAGCACTT-3'	
NtNtLEA5	F: 5'- TTGTTAGCAGGCGTGGGTAT-3'	
	R: 5'- CTCTCGCTCTTGTTGGGTTC-3'	
NtPOD	F: 5'- GCTGTTCGACGAGTTGTTAA-3'	
	R: 5'- CTCTGGCTGAGTTGTTGTTGG-3'	
NtSOD	F: 5'-AGCTACATGACGCCATTTCC-3'	
	R: 5'- CCCTGTAAAGCAGCACCTTC-3'	
NtCAT	F: 5'- AAGAATGGCTCCGCAAGTTA-3'	
	R: 5'- GCCTAGCAATTCCAGAGTGG-3'	
NtActin	F: 5'-CAAGGAAATCACCGCTTTGG-3'	
	R: 5'-AAGGGATGCGAGGATGGA-3'	

Table S2 Molecular characteristics of potato PYL family genes.

Name	Gene ID	Transcript ID	Protein ID	MW (Da)
StPYL1	PGSC0003DMG400000215	PGSC0003DMT400000606	186	20,793.66
StPYL2	PGSC0003DMG400010238	PGSC0003DMT400026521	191	21,587.47
StPYL3	PGSC0003DMG400020606	PGSC0003DMT400053119	201	22,158.08
StPYL4	PGSC0003DMG400020599	PGSC0003DMT400053102	201	22,158.08
StPYL5	PGSC0003DMG400020607	PGSC0003DMT400053120	201	22,158.08
StPYL6	PGSC0003DMG400009108	PGSC0003DMT400023511	185	20,958.80
		PGSC0003DMT400023512	154	17,274.54
StPYL7	PGSC0003DMG400027118	PGSC0003DMT400069734	208	22,885.80
StPYL8	PGSC0003DMG400023949	PGSC0003DMT400061535	218	23,996.03
		PGSC0003DMT400061536	218	23,996.03
StPYL9	PGSC0003DMG400002100	PGSC0003DMT400005378	213	23,716.28
StPYL10	PGSC0003DMG400029952	PGSC0003DMT400077008	188	21,082.03
StPYL11	PGSC0003DMG400017514	PGSC0003DMT400045156	231	25,285.95
StPYL12	PGSC0003DMG400012155	PGSC0003DMT400031686	189	21,572.67
StPYL13	PGSC0003DMG400029194	PGSC0003DMT400075056	214	23,368.31
StPYL14	PGSC0003DMG400015897	PGSC0003DMT400041080	207	22,815.81
StPYL15	PGSC0003DMG400011033	PGSC0003DMT400028658	213	23,496.32
StPYL16	PGSC0003DMG400005016	PGSC0003DMT400012887	177	20,186.94
		PGSC0003DMT400012888	177	20,186.94
StPYL17	PGSC0003DMG400029300	PGSC0003DMT400075340	190	21,242.03
StPYL18	PGSC0003DMG402001494	PGSC0003DMT400003774	161	17,354.7
StPYL19	PGSC0003DMG401001493	PGSC0003DMT400003773	161	17,887.22
StPYL20	PGSC0003DMG400023435	PGSC0003DMT400060243	163	18,140.83
